# A Preliminary Study on the Release of Bioactive Compounds from Rice Starch Hydrogels Produced by High-Pressure Processing (HPP)

**DOI:** 10.3390/gels9070521

**Published:** 2023-06-27

**Authors:** Anna D’Aniello, Katerina Koshenaj, Giovanna Ferrari

**Affiliations:** 1 ProdAl Scarl, c/o University of Salerno, 84084 Fisciano, Italy; a.daniello@prodalricerche.it; 2 Department of Industrial Engineering, University of Salerno, 84084 Fisciano, Italy; kkoshenaj@unisa.it

**Keywords:** starch-based hydrogels, high-pressure processing, green tea extract, bioactive compounds, transdermal release

## Abstract

This work aimed to carry out a preliminary study on the release of bioactive compounds loaded into starch-based hydrogels produced by high-pressure processing (HPP). As a study case, the experiments were carried out on rice starch HPP hydrogels. Rice starch (20% *w*/*w*) and green tea extract (2% *w*/*w*), suspended in distilled water, were treated by HPP at processing conditions enabling starch gelatinisation, namely 600 MPa for 15 min at room temperature. Additional experiments were carried out on samples that were further loaded with glycerol (5% *w*/*w*). Gel formation was assessed by analysing the gelatinisation extent, structuring level, and swelling power of the samples. At the processing conditions utilised, stable hydrogels were obtained even in the presence of the extract and/or the glycerol in the starch suspension. As expected, the colour of the hydrogels formed was affected by the addition of green tea extract in the starch solution. HPP starch hydrogels were characterised by Fourier transform infrared spectroscopy (FT-IR) to determine the interactions between the different compounds utilised in the formulation. Moreover, the release kinetics of bioactive compounds from HPP rice starch hydrogels was evaluated using a vertical Franz diffusion cells system, simulating a transdermal pattern. The diffusion of bioactive compounds was measured spectrophotometrically and via HPLC analysis. A controlled release of bioactive compounds from the hydrogel structure was detected, suggesting that small molecules, such as polyphenols, positively interacted with the rice starch HPP hydrogel network that allowed a smooth and constant release of these bioactive compounds over time.

## 1. Introduction

The increasing attention of consumers on keeping their living standards as high as possible, particularly their health, as well as the occurrence of pandemic and climate changes has promoted, especially in young adults, more consciousness in their purchase decisions and habits [1].

For this reason, in recent years the demand for products based on natural ingredients, including foods, cosmetics, and pharmaceuticals, is constantly increasing and their health-related benefits are widely recognised [2]. Moreover, consumers of all ages are interested in keeping a youthful aspect (PwC Deals, 2020), thus the market for cosmetics has grown exponentially with worldwide annual expenditure projected to reach 10 billion dollars by 2026 [3]. 

The so-called cosmeceuticals, containing biologically active ingredients have shown a rising interest not exclusively to counteract skin aging but, more generally, to protect the skin, nails, and hair, and to provide defence against harmful environmental factors, including ultraviolet radiation and free radicals [4,5,6,7]. Among these up-trending materials, hydrogels which represent a commercially widespread group of versatile polymeric structures can be envisaged as appropriate structures for the controlled release and target delivery of associated ingredients because of their safety, biocompatibility, hydrophilicity, and biodegradability [8]. Various hydrogels from renewable sources have been synthesized using alginate, gelatine, starch, chitosan, their derivatives, and cellulose [9], starch being one of the most promising biomaterials due to its biodegradability, abundance, and availability, as well as its low-cost compared to other polymers [10]. Moreover, through physicochemical modification, starch can show good film-forming and emulsification properties, which are desirable characteristics for encapsulation [11]. Starch-based hydrogels are typically prepared using conventional methods [9], but the unreliable processing conditions, environmentally hazardous preparation techniques, and materials, long operation times, high energy consumption levels, and gel network defects, are some of the actual limitations that prevent their further utilisation and commercialisation [12].

In this framework, high-pressure processing (HPP) has been proven as a suitable technology to prepare stable starch-based hydrogels overcoming the main limitations related to the traditional preparation methods. High-pressure processing (HPP) is a non-thermal technology able to modify the properties or gelatinize starches or proteins, which was also proposed for the production of highly structured and functional hydrogels based on aqueous starch suspensions [13,14,15,16,17,18]. Therefore, high-pressure processing is an appropriate production method for obtaining starches with increased potential health benefits and retaining the eco-friendly nature of hydrogels [13,14]. The starch gelatinisation under pressure depends on several factors, the most well-studied being starch source, pressure level, processing time, as well as processing temperature [19,20].

Depending on the type of starch used, HPP hydrogels may show creamy or gummy structures with good stability, which can be easily functionalised. Natural extracts can be added in the formulation of such biopolymers, which can be considered smart carriers for the encapsulation of the bioactive compounds to design “green” cosmeceutical, pharmaceutical, or food products [21]. Agri-food by-products and residues, as cost-effective matrices, represent a suitable source of valuable natural compounds, such as proteins, polysaccharides, fibres, flavour, pigments, and, in particular, bioactive compounds [22]. Green tea is one of the herbal products that has received increased attention from the industrial sector in recent years [23,24,25] due to its high content of polyphenols, particularly catechins. With its global production expected to grow at a faster annual rate of 7.5%, the implementation of strategies to use green tea processing by-products has become a challenge that should be pursued [26]. The large amounts of valuable compounds that can be recovered can be utilised for various scopes [27]. Nevertheless, natural substances are very sensitive to external factors, such as light, temperature, and phase separation; therefore, the encapsulation of bioactive compounds in appropriate structures is essential for their utilisation in novel products with improved quality and specific features.

However, to the best of our knowledge, no studies have been carried out to prove the capacity of starch-based HPP hydrogel to incorporate and release bioactive compounds, whose determination is of utmost importance to formulate new products for topical use based on these novel structures. This work aimed to investigate the gel formation and physical properties of rice starch hydrogels when green tea extract was added to the formulation as well as the release kinetics of these bioactive compounds encapsulated into starch-based hydrogels produced by high-pressure processing (HPP). To this purpose, a Franz diffusion cells system was used to determine the in vitro dermal release of bioactives.

## 2. Results and Discussion

### 2.1. Gel Formation

The starch gelatinisation under pressure was evaluated by macroscopic assessment and determination of the efficiency index and swelling power. It is well known that the production of starch-based hydrogels under pressure is influenced by various factors such as formulation and HPP processing conditions [21,28,29]. Rice starch has an A-type spatial configuration of its crystalline structure [15]. The A-type starch has a scattered amylopectin branching structure, which is more flexible, and therefore allows the rearrangement of double helices to generate a channel in which water molecules are included under pressure, resulting in smoother structures [20,30].

**Figure 1:**
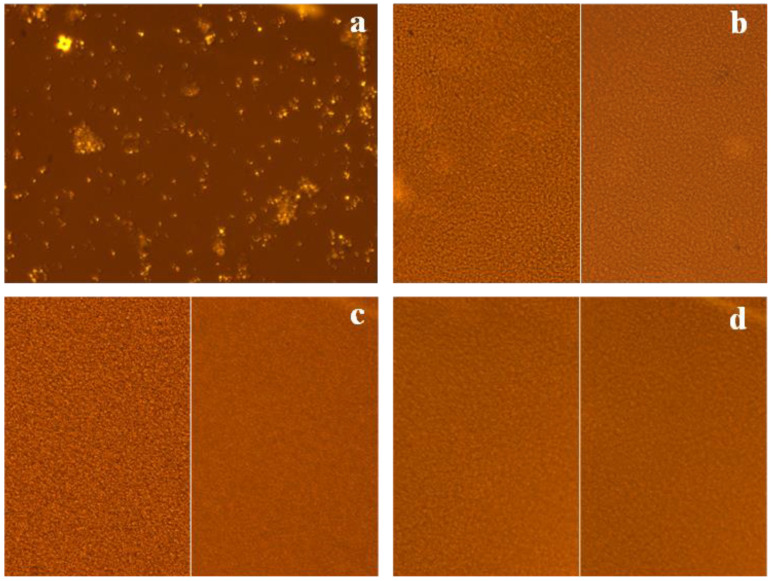
Micrographs of samples: birefringence of rice suspension (**a**); rice starch hydrogel, as control, under normal (left) and polarised (right) light (**b**); rice starch hydrogel loaded with 2% (*w*/*w*) of green tea extract under normal (left) and polarised light (right) (**c**); rice starch hydrogel with green tea extract (2% *w*/*w*) and 5% (*w*/*w*) of glycerol, under normal (left) and polarised (right) light (**d**).

As shown in Figure 1b, rice starch-based HPP hydrogels had a very homogeneous and cream-like structure due to the high number of starch granules per gram [31]. Moreover, under the treatment conditions utilised in this work (600 MPa for 15 min), the control samples exhibited a complete loss of birefringence, which is the ability to double-refract polarised light. All the starch granules in their native form exhibit birefringence proportional to their crystalline structure [32] and its loss accounts for starch granule deformation due to their modifications occurring during gelatinisation [33,34]. In all hydrogel samples, as is presented in Figure 1b–d, including those loaded with green tea extract and with green tea extract and glycerol, no birefringence was detected. This indicates that a complete gelatinisation of the starch suspensions under the treatment conditions utilised was likely to occur (Figure 1). Furthermore, the efficiency index values showed that a highly structured hydrogel was formed for all formulations, as reported in Table 1.

The swelling power, which is a property related to the length distribution of the amylopectin chain, the branching pattern, and the molecular weight [35,36] and accounts for the water-holding capacity of starch, was determined for all hydrogel samples. This parameter has been generally used to highlight the differences among various types of starches [37] and indicates the gel formation extent in the HPP-treated samples. The control samples exhibited higher swelling capacity compared to the loaded hydrogels, as shown in Table 1. The reduction in swelling power could be attributed to the limited swelling of starch granules due to their structure or composition. Since swelling is a property of amylopectin, the limited swelling detected in loaded samples could be due to the interaction between green tea extract compounds and amylopectin [38]. Indeed, in the green tea extract added to the formulations, a high sugar content was detected (Table 2), which affects the swelling of starch granules and the rheological properties of the suspensions [39]. Moreover, it has been demonstrated that most of the polyphenols present in green tea extract, containing phenolic hydroxyl groups, could connect with the starch molecules by non-covalent interaction and, at the molecular level, through the formation of V-type inclusion complexes and non-inclusion complexes [40]. The addition of polyphenols could accelerate HPP-induced gelatinisation and change the structural and physical properties of starch, enhancing the homogeneity of the final hydrogel [41]. Likewise, the addition of glycerol, which is the main moisturiser used in cosmetics and well-studied for its ability to modify the starch properties [42,43,44], caused a decrease in the swelling power of the starch granules by reducing the interaction between starch and water; hence the swelling in control samples is more intensive [45].

The results showed that rice starch-based hydrogels can be used as smart carriers of biocompounds to manufacture products where these cream-like structures are required.

### 2.2. Colour Measurements

**Figure 2:**
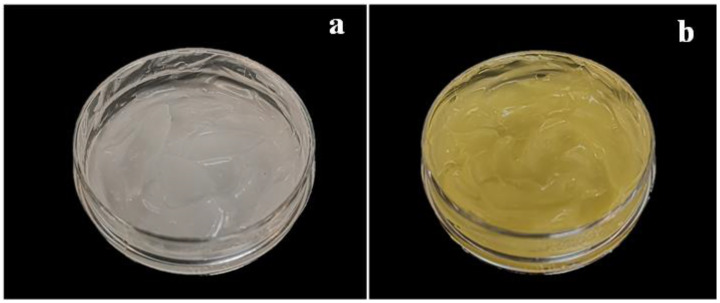
Picture of rice starch-based hydrogel (**a**) and green tea extract-loaded hydrogel (**b**).

Figure 2 shows pictures of rice starch-based hydrogel (control) and green tea extract-loaded hydrogel. Colour measurements were carried out to compare the appearance of the samples. Data reported in Table 3 showed that all HPP hydrogels prepared in this study have positive L* values, indicating that white and light components were predominant in all cases. These findings are further confirmed by WI values. Concerning the a* parameter, all hydrogels showed a tendency to greenness. The green is particularly related to the presence of chlorophyll, a natural pigment responsible for the green colour of plants and vegetables [46]. The negative value of the parameter b* in the control sample indicated its tendency to blueness, while the loaded hydrogels showed a high tendency to yellowness because of the pigments contained in the green tea extract. The yellowness is particularly related to the presence of β-carotene, a pigment abundant in plants [47].

To better understand if the colour differences among the hydrogels obtained, which are affected both by the loaded extract and starch source, could be detected by the human eye, the values of the parameter ΔΕ_ab_* were calculated. The human eye perceives the colour differences if ΔE_ab_* values are >3 [48]. It should be underlined that, as predictable, the results of the instrumental colour measurements support the results of the macroscopic evaluation. In all cases, when the addition of bioactive compounds changed, the colour profiles of samples were perceived by the human eye and compared to the controls. The value of colour difference ΔE_ab_* was high.

### 2.3. FT-IR Spectra Determination

**Figure 3:**
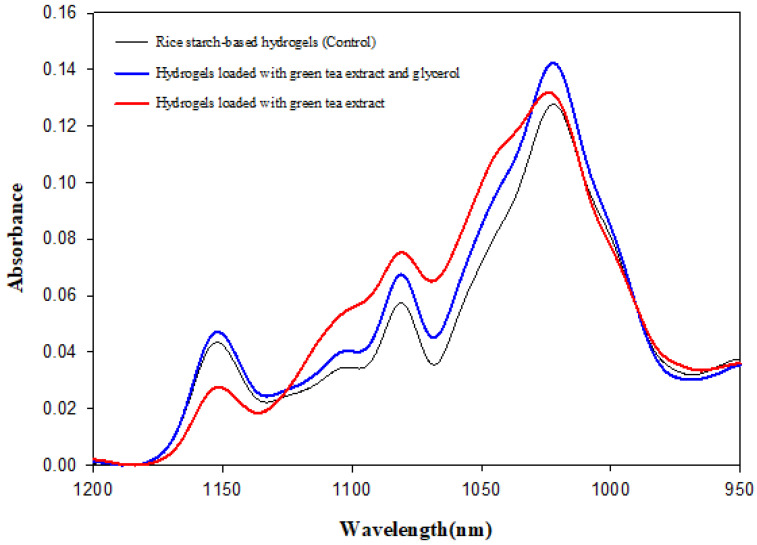
FT-IR spectra of hydrogels obtained at 600 MPa for 15 min.

FT-IR was used to determine the interactions between starch and water, as well as the additional compounds in the formulation, namely green tea extract and glycerol. Indeed, FT-IR is an important technique that can be used to determine organic compounds (proteins, carbohydrates, and lipids), including the chemical bonds between them [49]. In addition, it provides useful information for the characterisation of the gel structure [50,51]. In HPP hydrogels, polymer chains are cross-linked by the physical noncovalent interactions, and crystalline segments and hydrophobic interactions produce molecular connections with different strengths and stability [52]. The FT-IR spectra of the rice starch-based hydrogel (control) and the loaded hydrogels are shown in Figure 3. The bands of the infrared spectrum characteristic of starch gelation can be detected in the wavelength range 950–1200 cm^−1^. These bands mainly account for the C-O stretching of the ring and the linkage of C-O-C and COH groups, and their position is similar in all carbohydrates because the corresponding vibrational modes are unlikely to be significantly affected by polymer formation [53,54].

In this study, the changes in the intensity of the peaks in the above-mentioned sensitive starch region were observed. Based on the data reported in Figure 3, all spectra showed characteristic absorption peaks at 1023, 1080, and 1156 cm^−1^. The intense bands at 1156 cm^−1^ are assigned to the asymmetrical stretching vibrations C-O-C and the bands at 1080 and 1023 cm^−1^ are assigned to the stretching vibrations C-O [55,56]. Moreover, no new peak appeared in the spectra of hydrogels loaded with green tea extract and glycerol, indicating that no chemical interactions occurred between components via covalent bonding [57]. The absorbance peaks are related to the absorption bands of amorphous and crystalline regions and the number of ordered structures [58]. Thus, the absorption peaks have been identified as indicators of starch gelation [59], among which the amplitude of the peak at 1022 cm^−1^ indicates amorphous structures [51]. Hydrogels loaded with green tea extract and glycerol showed the highest absorption peaks at 1022 cm^−1^, accounting for a highly structured profile. As already discussed by other authors [58,60], higher absorption peaks correspond to higher structured profiles as also confirmed by analysing the results of rheology and texture measurements. Moreover, the addition of glycerol as an active moisturiser in gels has been proven as a suitable strategy to increase the stability and mechanical properties of hydrogels [43]. Thus, it can be concluded that loading green tea extract and glycerol in hydrogel formulation allowed for obtaining stronger and denser networks because of the stronger intermolecular and intramolecular interaction (Van der Waals forces, electrostatic, and hydrogen bonding), which are likely to occur.

### 2.4. In Vitro Transdermal Release by Franz Diffusion Cells

The design and development of the in vitro study of the transdermal release of bioactive compounds loaded into starch-based hydrogels was achieved through the optimisation of the diffusion conditions of the pure green tea extract and the results obtained were more fitted by applying different kinetic release models.

#### 2.4.1. Preliminary Diffusion Study

In a preliminary phase, several parameters were evaluated to determine the optimal diffusion conditions that are influenced by the solubility of the bioactive compounds present in the green tea extract. In particular, the sink conditions should be reached and maintained, meaning that the backflow from the acceptor phase can be considered equal to zero [61]. In this perspective, the choice of the proper receptor fluid, the release time, and the time intervals for sample withdrawal are crucial to creating suitable sink conditions.

More specifically, according to the studies found in the literature [62,63,64], four different fluids were tested, as reported in Table 4. The release behaviour of the pure green tea extract was determined utilising five Franz diffusion cells for 8 h. The release time was set based on the usage instructions of most of the topical creams available on the market. Moreover, to satisfy the sink conditions, the withdrawals were made at specific time intervals, initially after 30 min and then after every hour.

The appropriate receptor fluid was selected according to the cumulative amount of polyphenols and antioxidants released at the end of the test, and the possible presence of foam and residues in the cell, as summarised in Table 4. It was observed that the release of bioactive compounds over time was affected by changing the composition and the pH of the acceptor fluid. Therefore, based on these considerations, the artificial human sweat was selected as the most suitable receptor fluid mimicking the skin conditions and providing reliable data (TPC: 8.4%, AA: 13.7%; homogeneous and transparent appearance of the solution in the cell).

#### 2.4.2. Release of Bioactive Compounds

The in vitro dermal release of bioactive compounds loaded into rice starch-based hydrogels was investigated utilising a Franz diffusion cells system for 8 h with artificial human sweat as receptor fluid. 

**Figure 4:**
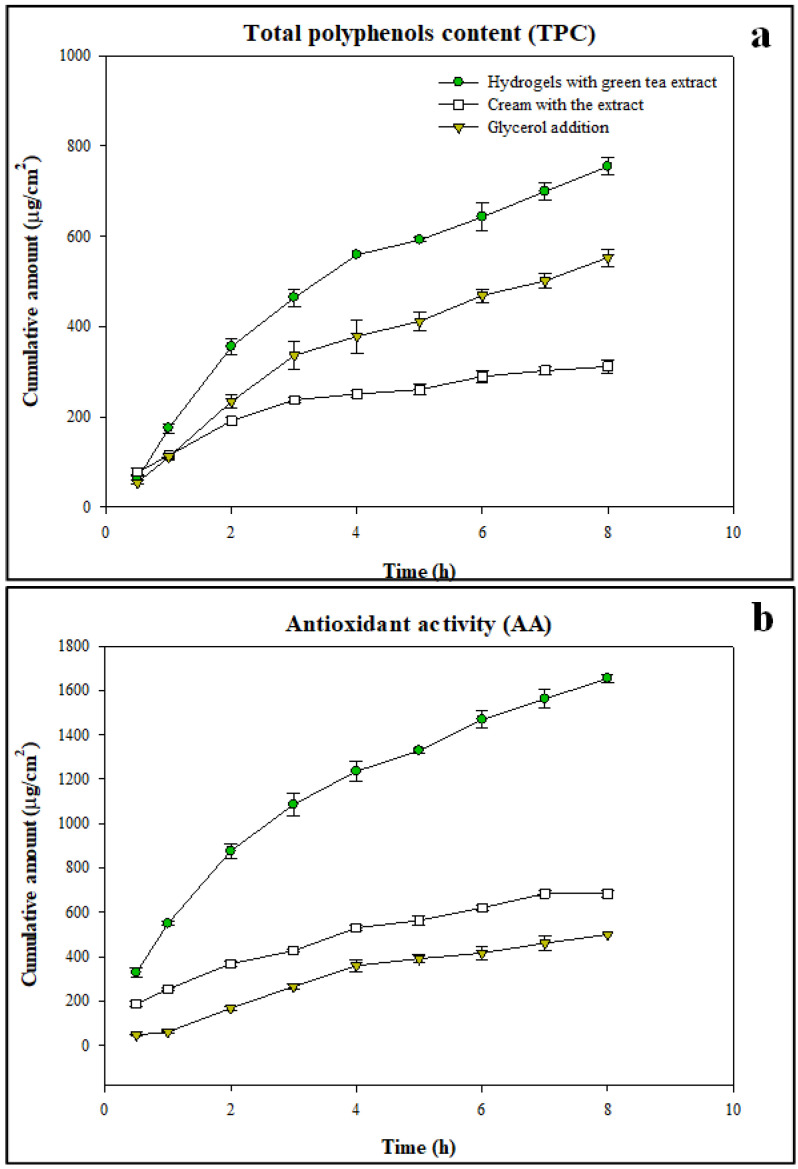
Release profiles of total polyphenols (**a**) and antioxidant compounds (**b**) from starch-based hydrogels, from commercial cream, and in the presence of glycerol.

A controlled and constant release of bioactive compounds from the hydrogel structure over time was recorded, as shown in Figure 4. It is known that green tea polyphenols interact with starch to form starch-polyphenols inclusion complexes through hydrophobic interactions [65] and this was confirmed by analysing the results of FT-IR measurements where no new peaks were detected. The bioactive compounds of green tea extracts were interacting with the rice starch hydrogel leading to their effective encapsulation inside the structured materials which, in turn, allowed their smooth release. Moreover, the observed trend was confirmed by HPLC analyses of the receptor fluid, which was withdrawn at different times from the Franz cells. An increasing concentration of the identified polyphenols released was detected as reported in Table 5, with epicatechin having the highest concentration. Additionally, the bioactive release kinetics was described using zero-, first-, Higuchi, and Korsmeyer–Peppas models [66,67]. The data were best fitted with the Korsmeyer–Peppas model (total polyphenols: R^2^ = 0.9873, *n* = 0.61; antioxidants: R^2^ = 0.9955, *n* = 0.50). Considering the *n*-values, it was possible to conclude that the mechanism of biocompound release from HPP rice starch-based hydrogels was diffusion and swelling; however, the diffusion was predominant due to the polymer chain relaxation process [68]. This is coherent with our findings regarding the reduction of the swelling capacity of hydrogels observed in the presence of green tea extract compounds, as reported and discussed in Section 2.1.

To better understand the release behaviour of gels, green tea extract was added to the commercial cream (a neutral emulsion of water in oil) at the same concentration (2%) used in rice-starch hydrogels. The sample was loaded in the Franz cells system, as also depicted in Figure 4, and the release of biocompounds over time was determined. In this case, the release kinetics data are better described by the Higuchi model (total polyphenols: R^2^ = 0.9782; antioxidants: R^2^ = 0.9783), assessing that the release mechanism of biocompounds was driven by diffusion. Rice starch HPP hydrogels showed a noticeably greater release of biocompounds compared to the cream probably due to the strong network characterising the hydrogel structure and its swelling capacity acting as permeation enhancers. In addition, Figure 4 also shows the trend with time of the cumulative amount of polyphenols and antioxidants released from the hydrogels loaded with the extract and in the presence of glycerol (5% *w*/*w*). The release kinetics, in this case, are better described utilising the Korsmeyer–Peppas model (total polyphenols: R^2^ = 0.9917, *n* = 0.64; antioxidants: R^2^ = 0.9863, *n* = 0.77), with diffusion and polymer chain relaxation process rates comparable. However, a slower release of the bioactives from this structure was observed due to the combined presence of glycerol and green tea extract that hindered the swelling capacity of the hydrogel, as discussed in Section 2.1. Moreover, hydrogels loaded with green tea extract and glycerol show a highly structured profile, as highlighted in the FT-IR spectra of Figure 3, which could also explain the lower release rate of bioactive compounds detected. Therefore, while the incorporation of glycerol in the formulation allowed to obtain a more stable and denser structure, it resulted in a further decrease in the bioactives release rate. The calculated values of the total polyphenols flux released were 86.05 μg/cm^2^/h, 29.24 μg/cm^2^/h, and 63.46 μg/cm^2^/h from hydrogels loaded with the green tea extract, hydrogels loaded with the green tea extract and glycerol, and cream, respectively. The flux value was higher for hydrogels loaded with the extract, followed by the hydrogels loaded with both the extract and glycerol as well as the cream.

These results further highlighted that rice starch-based hydrogels loaded with bioactive compounds such as green tea extract represent suitable and effective carriers for their controlled release in topical applications.

Although the presence of glycerol did not affect the gel formation, its addition needs to be optimised to individuate the optimal glycerol percentage enabling it to obtain the desired performances of the hydrogel structure. Indeed, glycerol has been included for many years in topical preparations thanks to its hydrating effect [69]; therefore, its inclusion in the hydrogel structure represented the first approach for the design of new cosmetics and cosmeceuticals. However, further work is needed to better understand fundamental aspects regarding the characteristics of HPP starch-based hydrogels, particularly, the relationship between the gel strength and the behaviour during the release of the bioactive compounds included in the formulation.

## 3. Conclusions

This work demonstrated that the utilisation of HPP starch-based hydrogels as smart carriers of bioactive compounds can be envisaged, which is a good starting point for the design of topical products. The results obtained highlighted that the addition of green tea extract in the formulation of rice starch suspensions did not hinder the gelation process under high pressure and did not affect the structural integrity, homogeneity, and cream-like appearance of the hydrogels. A highly structured hydrogel was formed when the green tea extract was incorporated at a concentration of 2% (*w*/*w*), which is the maximum allowed concentration of this extract in topical products. A controlled release over time of the bioactive compounds from the structure of the hydrogels was detected, highlighting their effective encapsulation inside these structured materials. The addition of glycerol at a percentage of 5% translated into an improvement of the structural profile of the hydrogel, as confirmed by FT-IR spectra, while a decrease in biocompound release was observed. More work is needed to better understand the relationship between the strength of the hydrogels and their behaviour during release over time of the biocompounds included in their formulation. Moreover, the physical and microbiological stability of these structured materials during shelf life should be also assessed.

## 4. Materials and Methods

### 4.1. Materials

Rice starch powder (S7260) (17.7% amylose content) was supplied by Sigma Aldrich (Steinheim, Germany). Distilled water was used to prepare all starch solutions. Green tea extract was obtained from industrial agri-food residues using extraction processes assisted by non-thermal technologies. The commercial cream (aqua, cetearyl alcohol, ethylhexyl stearate, paraffinum liquidum, glycerine, glyceryl stearate, ceteareth-20, cera alba, propylene glycol, xanthan gum, dimethicone, phenoxyethanol, ethylhexylglycerin), which represented the control, was purchased from AIESI Hospital Service (Naples, Italy). All other chemicals and reagents were of analytical grade or superior.

### 4.2. Sample Preparation and Hydrogel Production

A suspension of rice starch powder and distilled water at a concentration of 20% (*w*/*w*) was used to prepare the hydrogels, based on the results of previous studies [21]. Green tea extract was added to the suspension to reach a final concentration of 2% (*w*/*w*). The latter, according to [70], is the maximum allowed concentration of this bioactive in cosmetic applications. Additional samples were prepared by adding to the starch solution loaded with green tea extract 5% of glycerol (*w*/*w*), which is able to promote hydrogel formation by HPP and allows the reinforcement of the rheological and textural properties of the structures formed [43].

For each sample, 3 g of the starch suspension was thoroughly mixed and vacuum-packed in flexible bags (polymer/aluminium/polymer film OPP30-A19-LDPE70). The suspensions were agitated until full homogenisation to prevent particle sinking and then treated under pressure in a laboratory-scale high-pressure unit (U111, Unipress, Warsaw, Poland) previously described elsewhere [71]. Briefly, the equipment is provided with five high-pressure Cu-Be alloy vessels (inner volume 9 mL) working in parallel, submerged in a thermostatic bath containing silicon oil (M60.115.05, #85321, Novo-direct, Bagsvaerd, Denmark), and can operate at pressures up to 700 MPa and temperatures between −40 °C and 100 °C. 

Based on the results of previous studies [43], the pressure applied in this investigation to achieve the complete gelatinisation of the samples was set at 600 MPa, with a processing time of 15 min, at room temperature (25 °C). All experiments were performed in triplicate. Treated samples were stored at ambient temperature until further analyses.

### 4.3. Gel Formation Determination

Gel formation was assessed by determining the gelatinisation extent, structuring level, and swelling power of the samples.

#### 4.3.1. Microscopy Evaluation

The degree of gelatinisation was evaluated by measuring the loss of birefringence of the starch granules using an optical inverted microscope (Nikon Eclipse, TE 2000S, Nikon Instruments Europe B.V., Amsterdam, The Netherlands) with a polarisation filter and a 20× objective coupled to a DS Camera Control Unit (DS-5M-L1, Nikon Instruments Europe B. V., Amsterdam, The Netherlands) for image acquisition and analysis. Before observation, a small amount of the sample was spotted on a microscope slide and covered with cover glass.

#### 4.3.2. Efficiency Index

The gelatinisation level of samples under HPP conditions was assessed by evaluating using Equation (1) the efficiency index (EI), as proposed by Larrea-Wachtendorff [21]. The EI represents a crucial parameter that specifically indicates the drained weight of the structural material.

(1) $$\mathrm{EI}=\frac{\mathrm{Hydrogel}\;\mathrm{formed}\;\left(g\right)}{\mathrm{Starch}\;\mathrm{suspension}\;\mathrm{before}\;\mathrm{HPP}\;\mathrm{treatment}\;\left(g\right)}$$

#### 4.3.3. Swelling Power

The swelling power was determined by modifying the method described by Kusymayanti [72] according to Larrea-Wachtendorff [21]. HPP-treated samples were centrifuged (PK130R, ALC, Winchester, Virginia) at 1351× *g* for 10 min and the pellet was weighed before and after drying for 6 h at 105 °C. The swelling power ratio, evaluated by Equation (2), is defined as the weight of the wet pellet over the dry weight of the starch in the hydrogel samples:

(2) $$\mathrm{SP}\;\left(g/g\right)=\frac{\mathrm{Weight}\;\mathrm{of}\;\mathrm{the}\;\mathrm{wet}\;\mathrm{pellet}\;\left(g\right)}{\mathrm{Weight}\;\mathrm{of}\;\mathrm{the}\;\mathrm{dried}\;\mathrm{hydrogel}\;\mathrm{sample}\;\left(g\right)}$$

### 4.4. Colour Measurements

The lightness (L*), redness (a*), and yellowness (b*) of the HPP hydrogels were measured against a white background using a colorimeter CR-400 (Konica Minolta Inc., Tokyo, Japan).

The colour difference (ΔΕ_ab_*) was determined using Equation (3):

(3) $$\Delta E_{\mathrm{ab}}*\;=\sqrt{{\left(\Delta L^{*}\right)}^{2}+{\left(\Delta a^{*}\right)}^{2}+{\left(\Delta b^{*}\right)}^{2}}$$

where ΔL*, Δa*, and Δb* are the colour differences between the control and loaded hydrogels samples in L*, a*, and b*, respectively.

The whiteness index (WI) was evaluated using Equation (4), as reported by Kaur [73].

(4) $$\mathrm{WI}=100-\sqrt{{\left(100-L^{*}\right)}^{2}+{\left(a^{*}\right)}^{2}+{\left(b^{*}\right)}^{2}}$$

### 4.5. Fourier Transform Infrared Spectroscopy (FT-IR) Measurements

Fourier Transform Infrared Spectroscopy (FT-IR) was used to characterise the presence of specific chemical groups in the hydrogels. Each sample (control and loaded hydrogels) was analysed using an FT/IR-400 spectrometer (Jasco Corporation, Kyoto, Japan). A total of 128 scans were obtained for each measurement at a resolution of 4 cm^−1^. The FT-IR spectra of the samples were recorded at wavelengths ranging from 1200 to 950 cm^−1^. The resulting starch-averaged spectrum was smoothed to remove any potential noise using a fifteen-point adaptive smoothing function. Subsequently, the baseline modification and a normalised function were applied.

### 4.6. Release of Bioactive Compounds

The determination of the release of bioactive compounds from hydrogels was performed on a system of five vertical Franz diffusion cells operating in parallel (SES GmbH-Analysesysteme, Bechenheim, Germany) using dialysis tubing cellulose membranes (MWCO = 14 kDa, Sigma-Aldrich, Steinheim, Germany). The membranes (*n* = 5) were previously soaked in the receptor fluid for 24 h and then placed between the donor and the receptor compartments. A volume of 5 mL of solution was used as the acceptor phase and the diffusion area was 0.64 cm^2^. The receptor phase was kept at 32.5 ± 0.5 °C in thermostatic conditions with an external jacket and agitated with a magnetic stirrer. Then, 1 mL of the hydrogel containing the bioactive compound was loaded on the membrane. At given time intervals aliquots of 0.2 mL were collected from each cell simultaneously and replaced with fresh receptor fluid [74]. To address the mechanism of bioactive release from hydrogels, the same procedure was repeated by using the green tea extract cream, obtained by manually mixing the green tea extract and a commercial cream base, as described by Mendes de Moraes [66]. Bioactive compounds released were quantified spectrophotometrically using a V-650 UV spectrophotometer (Jasco Instruments, Easton, MD, USA), and the antioxidant activity (AA) and total polyphenols content (TPC) were determined. The polyphenols were identified via High-Performance Liquid Chromatography—Photodiode Array Detection (HPLC-PDA).

#### 4.6.1. Antioxidant Activity Determination

The antioxidant activity (AA) of the samples was determined using the ferric reducing antioxidant power (FRAP) assay in accordance with the modified variant of Benzie and Strain’s method [75]. In brief, 2.5 mL of freshly prepared FRAP working solution was added to 0.5 mL of the diluted sample, and the mixture was incubated for 10 min at room temperature. The absorbance measurement of the reacting mixture was then carried out at 593 nm using pure FRAP reagent as blank. The AA was expressed as the concentration of ascorbic acid in ppm (C_AA_) according to Equation (5):

(5) $$C_{\mathrm{AA}}=176.1\times \frac{A_{593}+0.0330063}{9.4405}$$

where A_593_ is the absorbance measured at 593 nm.

#### 4.6.2. Total Polyphenol Content Determination

The concentration of polyphenols was measured by the method of Folin–Ciocalteau as described by Bobinaitė [76], with slight modifications. Briefly, 0.5 mL of the diluted sample, 2.5 mL of 10% (*v*/*v*) Folin–Ciocalteau reagent, and 2 mL of sodium carbonate (7.5%, *w*/*v*) were mixed and incubated in the dark for 60 min. The absorbance of the reacting mixture was then measured at 765 nm against the blank (a mixture of 0.5 mL of distilled water, 2.5 mL of diluted Folin–Ciocalteau, and 2 mL of sodium carbonate solution). The total polyphenols content (TPC), expressed as the concentration of gallic acid in ppm (C_GA_), was evaluated according to Equation (6).

(6) $$C_{\mathrm{GA}}=\frac{A_{765}}{0.0119488}$$

where A_765_ is the absorbance measured at 765 nm.

#### 4.6.3. HPLC-PDA Analysis

The loaded hydrogel samples were subjected to HPLC-PDA analysis using a Waters 1525 Separation Module linked to a Waters 2996 photodiode array detector (Waters Corporation, Milford, MA, USA), in accordance with a modified method reported by Carpentieri [77]. The detected compounds were separated analytically in a Waters Spherisorb C18 reverse-phase column (5 μm ODS2, 4.6 250 mm, Milford, MA, USA).

The mobile phase consisted of phosphoric acid (0.1%, eluent A) and methanol (100%, eluent B). Analytical separation was carried out by the following gradient elution: 0–30 min from 5% B to 80% B, 30–33 min 80% B, and 33–35 min from 80% B to 5% B. The injection volume and the flow rate of the mobile phase were 5 µL and 0.8 mL/min, respectively. The signal was provided for the quantification of each compound at the wavelength of maximum absorbance. All commercial standards were dissolved into distilled water to generate 6-point standard calibration curves (R^2^ = 0.999). The results were represented in terms of mg of the target components (gallic acid, catechin, epicatechin, and rutin) per L of the withdrawn sample.

#### 4.6.4. Release Kinetics Models

The kinetics of the in vitro release was defined through linear regression analysis. To this purpose, the results obtained via spectrophotometric determinations were fitted with mathematical models, such as zero-order (Equation (7)), first-order (Equation (8)), Higuchi’s model (Equation (9)), and Korsmeyer–Peppas model (Equation (10)) equations [66,67].

(7) $$Q_{t}={\;Q}_{0}+{\;k}_{1}t$$

(8) $${\mathrm{lnQ}}_{t}={\mathrm{lnQ}}_{0}+\frac{k_{2}t}{2.303}$$

(9) $$Q_{t}={\;k}_{3}t^{0.5}$$

(10) $$\ln\left(\frac{M_{T}}{M_{\infty }}\right)={\mathrm{lnk}}_{4}+\mathrm{nlnt}$$

where Q_t_ is the cumulative amount of bioactive compounds released at time t; Q_0_ is the initial amount of bioactive; k_1_, k_2_, k_3_, and k_4_ are the release kinetic constants for the above equations; M_T_ is the amount of bioactive released at time t, M_∞_ is the amount of bioactive released at infinite time, and n is the release exponent.

### 4.7. Statistical Analysis

All the experiments and the analyses of the produced hydrogels were carried out in triplicate, and the results were presented as means ± standard deviations. One-way analysis of variance (ANOVA) was used to assess differences between mean values using the statistical software SPSS 20 (SPSS IBM, Chicago, IL, USA). Tukey test was performed to identify statistically significant differences (*p* < 0.05).

## Figures and Tables

**Table 1:** Efficiency index and swelling power of hydrogels obtained at 600 MPa for 15 min.

HPP Treated Samples	Gel Formation Parameters	
	Efficiency Index	Swelling Power (g/g_dry starch_)
Rice starch hydrogels (control)	$1.00\;\pm \;0.00{\;}^{a}$	$5.91\;\pm \;0.03{\;}^{c}$
Rice starch hydrogels loaded with green tea extract	$1.00\;\pm \;0.00{\;}^{a}$	$5.32\;\pm \;0.11^{\;b}$
Rice starch hydrogels loaded with green tea extract and glycerol	$1.00\;\pm \;0.00{\;}^{a}$	$4.11\;\pm \;0.08{\;}^{a}$

Different letters in the same column represent significant differences at *p* < 0.05 probability level.

**Table 2:** Chemical composition of the green tea extract (dry weight basis).

Ash (g/100 g_DW_)	Protein (g/100 g_DW_)	Fat (g/100 g_DW_)	Carbohydrate (g/100 g_DW_)	Total Dietary Fiber (g/100 g_DW_)
2.1	9.7	0.5	61.9	25.3

**Table 3:** Colour parameters of rice starch HPP hydrogels (control) and loaded hydrogels with green tea extract obtained at 600 MPa for 15 min.

HPP Hydrogel	Colour Parameters				
	L*	a*	b*	WI	ΔE_ab_*
Control hydrogel	45.6 ±$\;0.32^{\;b}$	−0.07 ± $0.08{\;}^{b}$	−2.02 ± $0.08^{\;a}$	-	-
Hydrogel with green tea extract	39.84 ± $0.34^{\;a}$	−1.21 ± $0.02^{\;a}$	22.22 ± $0.4^{\;b}$	36.3 ± 0.69	24.5 ± 0.48

Different letters in the same column represent significant differences at *p* < 0.05 probability level.

**Table 4:** Comparison of the different receptor fluids on the release behaviour of the pure green tea extract.

Receptor Fluid	The Cumulative Amount of Bioactive Released	Inference
Phosphate buffer solution (PBS), pH 7.4	TPC: 5.4% AA: 8.4%	Foam heap during the time of release
PBS/10% of ethanol (1:1)	TPC: 7.9% AA: 8.5%	Evaporation issues
Citrate-phosphate buffer, pH 5.5	TPC: 6.9% AA: 5.7%	Passage of extract residues
Artificial human sweat	TPC: 8.4% AA: 13.7%	Homogeneous and transparent solution

**Table 5:** The concentration of the main phenolic compounds at different times (30 min, 2 h, 4 h, 8 h) identified through HPLC analysis and released from starch-based hydrogels.

Sample	Gallic Acid (mg/L)	Catechin (mg/L)	Epicatechin (mg/L)	Rutin (mg/L)
30 min	/	4.10	13.95	0.72
2 h	1.09	6.62	32.18	1.19
4 h	1.21	6.17	52.80	1.28
8 h	1.50	6.80	76.63	1.82

## Data Availability

The data presented in this study are available in the article.
